# An atypical case of Klippel-Trénaunay syndrome presenting with crossed-bilateral limb hypertrophy and postaxial polydactyly: a case report

**DOI:** 10.1186/s12887-019-1480-0

**Published:** 2019-04-06

**Authors:** Rawan M. Al-Najjar, Rafael Fonseca

**Affiliations:** 1 Department of Pediatrics, Texas Children’s Hospital, Houston, Texas USA; 2 Division of Neonatology, Department of Pediatrics, University of Texas Medical Branch at Galveston, Galveston, Texas USA

**Keywords:** Port wine stain, Hypertrophy, Vascular malformation, Klippel-Trénaunay syndrome, Cross-bilateral limb involvement, Polydactyly

## Abstract

**Background:**

Klippel-Trénaunay syndrome (KTS) is a rare congenital condition characterized by the clinical triad of capillary malformations (port wine stains), varicose veins with or without venous malformations, and bony and/or soft tissue hypertrophy.

**Case presentation:**

Here we report the first case of a one-day-old male with KTS presenting with crossed-bilateral limb hypertrophy and post-axial polydactyly.

**Conclusion:**

This case serves to highlight the variable presentation and multiple problems faced by patients with KTS and why multidisciplinary management is mandatory.

## Background

Klippel-Trénaunay syndrome (KTS) is a rare congenital disorder first described over a hundred years ago by the French physicians Klippel and Trénaunay [1]. KTS is usually recognized at birth but can present in older children and adults, and it is characterized by the clinical triad of capillary malformations (port wine stain), atypical venous malformations, and bony and/or soft tissue hypertrophy; any two of these features secures the diagnosis [2]. Vascular malformations are always present and usually (but not always) affect only one extremity, particularly the lower extremities [3]. KTS is usually sporadic, although familial cases are described suggesting that it can be inherited [4, 5]. Here we describe the first case of KTS presenting with crossed-bilateral limb hypertrophy and post-axial polydactyly.

## Case presentation

A one-day-old male Hispanic infant was delivered by normal spontaneous vaginal delivery at 40 weeks gestation with no complications. The mother’s pregnancy was complicated by anemia and polyhydramnios, but otherwise the mother had no notable environmental exposures and was healthy. A cystic malformation and possible teratoma of the cord were noted at the first ultrasound at week 23, which was at this time due to late maternal entry into antenatal care. Amniocentesis was performed and revealed a normal male karyotype. A second ultrasound in the third trimester revealed a large multicystic lesion in the left fetal body and significant swelling of the right leg and foot due to similar cystic masses. An MRI scan undertaken three weeks before delivery showed a multiseptate cystic mass in the left supraclavicular region, upper chest, and upper arm. The left lower extremity was asymmetrically smaller than the right lower extremity.

**Fig. 1:**
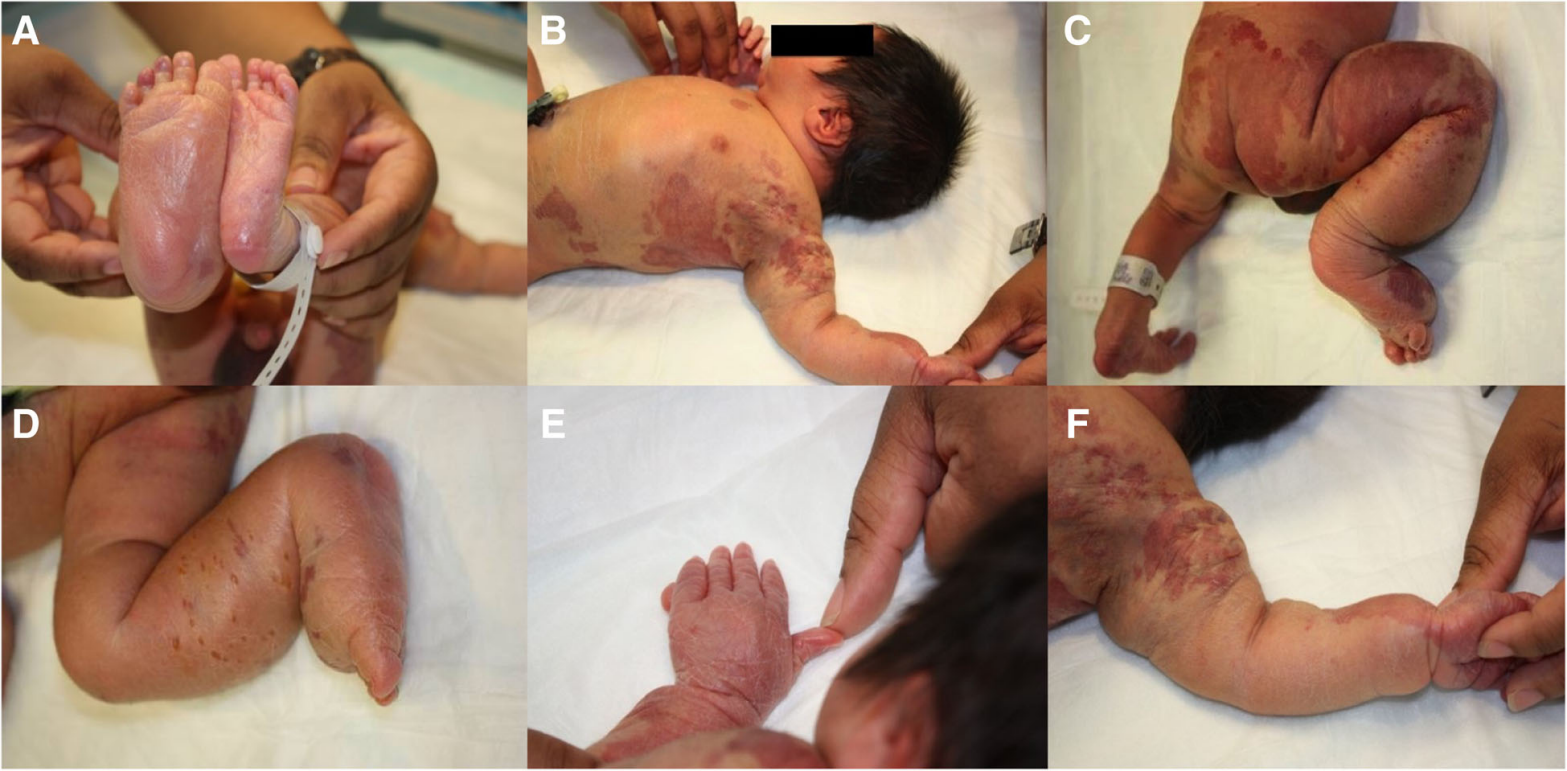
(**a**) A major discrepancy in the length and width of the lower limbs and hypertrophy of the right lower limb at day one of life. (**b**, **c**) Bullae and vesicular lesions on the trunk, port wine stains, and crossed-bilateral hypertrophy of the right lower limb and left upper limb at day one of life. (**d**) Right lower limb hypertrophy at day one of life. (**e**) Postaxial polydactyly of the left hand at day one of life. (**f**) Left upper arm hypertrophy and vesicular lesions at day one of life

The infant was admitted to the newborn nursery after birth with stable vital signs (weight 3585 g, respiratory rate 40, heart rate 150, SpO_2_ 98%, temperature 37.5 °C). The physical appearances are shown in Fig. 1A-F. There was obvious enlargement of the right lower extremity (Fig. 1A,C,D) and left upper extremity (Fig. 1B,F) and with numerous port wine stains on the chest, arms, lower back, and thighs (Fig. 1B,C,F). Bullae and vesicular lesions were also noted (Fig. 1B,F), as was polydactyly of the left hand (Fig. 1E). These features were compatible with KTS. There was no family history of the disorder.

A coagulation profile was significant for increased prothrombin time (17 s, reference 11–15 s) and high fibrinogen (370 mg/dl, reference 175–350 mg/dl), a profile often seen in newborns and that subsequently normalized the following day, ruling out intravascular coagulopathy. Head and abdominal ultrasounds ruled out internal arteriovenous malformations. Orthopedics assessed the right lower limb hypertrophy and recommended no intervention at that time with outpatient follow-up after discharge. Ophthalmic examination revealed a cutaneous hemangioma on the right upper eyelid, and outpatient follow-up was again advised.

The infant passed the hearing screen and was discharged from hospital in good clinical condition. Over the first year and a half, he developed normally, meeting all of his developmental milestones. Sclerotherapy was successfully conducted on some of his port wine stains, and the extra digit was removed without complication. A genetics evaluation revealed no family history of KTS, but there was a history of polydactyly in the maternal grandfather. Orthopedic review showed no change in the limb hypertrophy. However, he developed a new neck mass that was diagnosed as a lymphatic cyst by ultrasound and, at a year and a half, gross hematuria prompted a renal ultrasound that showed a cystic renal mass. Care was transferred to another hospital at that point.

## Discussion and conclusion

Klippel-Trénaunay syndrome is a congenital vascular abnormality that usually involves a single lower extremity and consists of the triad of vascular malformations, venous/lymphatic varicosities, and soft tissue and/or bony hypertrophy.

The prevalence of KTS is low or under-reported; however, it is thought to affect males and females equally with no racial predilection. The etiology of KTS is unknown, but the leading hypothesis is damage to the sympathetic nervous system resulting in dilatation and persistence of microscopic arteriovenous anastomoses in utero [6]. A color Doppler ultrasound should be performed for prenatal diagnosis of limb hypertrophy and to assess the underlying cause of any cystic lesion [7].

Most patients present with the complete clinical triad, with port wine stains and vascular malformations first appearing at birth and varicose veins usually appearing during infancy and progressing in adolescence [3]. Limb hypertrophy is often present at birth or during infancy and continues to grow until the child stops growing, although it may continue to progress over time. The hypertrophy is a result of soft tissue and/or bony overgrowth or lymphatic and venous malformations [3, 8].

This patient had multiple cystic masses which all were lymphatic malformations. Lymphatic malformation presents as localized or generalized overgrowth caused by micro- and macrocystic anomalies, sometimes in association with lymphedema. Often there is lymphatic swelling and fatty deposition on the contralateral foot. The lymphatic anomalies can also occur in the pelvis, bladder and lower gastrointestinal tract. Lymphatic cysts in the spleen are also common. Lymphatic malformation is documented by ultrasonography and/or MRI. Lymphography shows that lymphedema is the result of diminished number or absence of lymphatic channels.

Our patient had port wine stains on the trunk, back, and extremities and cross-bilateral hypertrophy of the right lower limb and left upper limb, which is unusual since limb involvement is most often unilateral (85%), sometimes bilateral (12.5%), and only rarely crossed-bilateral (2.5%) [9]. Varicose veins were not present on physical examination or imaging. Further, this patient had post-axial polydactyly of the left hand, which has only previously been described in four KTS cases [10–13], none with cross-bilateral limb involvement. Other limb anomalies including macrodactyly, syndactyly, clinodactyly, camptodactyly, ectrodactyly, and congenital hip dislocation have been reported in association with KTS [8]. However, this case represents the first KTS case with both cross-bilateral limb involvement and polydactyly. The patient had history of polydactyly in a maternal grandfather. Polydactyly can be inherited as well as forming part of the KTS syndrome [8], so it is unclear in this case whether the polydactyly was coincidental or genuinely part of the KTS spectrum.

KTS can cause significant morbidity from the vascular anomalies including deep venous thrombosis, bleeding, pulmonary embolism, stasis dermatitis, cellulitis, and limb enlargement that may require amputation. Patients also suffer from scoliosis and gait abnormalities related to limb hypertrophy. Therefore, KTS must be suspected, recognized, and appropriately managed in all infants with capillary malformations involving one or more extremity at birth.

There is currently no cure or definitive treatment for KTS. However, management should be multidisciplinary and aim to reduce the symptoms and complications of the disease. For instance, compression stockings can be used for varicose veins, and heel inserts can be used and are usually sufficient for leg length discrepancies of 1.5 cm or less, although surgical closure of the growth plate at the knee is sometimes needed to equalize the leg length. Corrective orthopedic braces can be used to prevent the development of vertebral deformities, especially scoliosis, in the presence of lower limb hypertrophy. Port wine stains are treated with pulsed dye laser therapy. KTS is generally not thought to be hereditary, and the risk of KTS in the offspring of parents with KTS is similar to the general population risk. However, KTS has been shown to belong to a spectrum of segmental overgrowth diseases caused by mutations in the *PIK3CA* gene; indeed, genetic analyses of KTS cases have shown that KTS is caused by mosaic activating mutations in *PIK3CA* [14, 15].

In conclusion, here we present a case of KTS with a unique constellation of signs: cross-bilateral limb involvement and polydactyly. This case not only serves as a review of the features and pathoetiology of the syndrome, but also highlights that patients must be followed up on a regular basis by a multidisciplinary team. As in our case, KTS can progress and new complications may arise that require multidisciplinary management. The prognosis of KTS is variable and depends upon the extent of disease and presence of complications.

## Abbreviations

KTS: Klippel-trénaunay syndrome
MRI: Magnetic resonance imaging
PIK3CA: phosphatidylinositol-4,5-bisphosphate 3-kinase catalytic subunit alpha
